# Optimizing viral load testing access for the last mile: Geospatial cost model for point of care instrument placement

**DOI:** 10.1371/journal.pone.0221586

**Published:** 2019-08-26

**Authors:** Sarah J. Girdwood, Brooke E. Nichols, Crispin Moyo, Thomas Crompton, Dorman Chimhamhiwa, Sydney Rosen

**Affiliations:** 1 Health Economics and Epidemiology Research Office, Department of Internal Medicine, School of Clinical Medicine, Faculty of Health Sciences, University of the Witwatersrand, Johannesburg, South Africa; 2 Department of Global Health, School of Public Health, Boston University, Boston, MA, United States of America; 3 EQUIP Zambia, Lusaka, Zambia; 4 Right to Care, GIS Mapping Department, Johannesburg, South Africa; Yeshiva University Albert Einstein College of Medicine, UNITED STATES

## Abstract

**Introduction:**

Viral load (VL) monitoring programs have been scaled up rapidly, but are now facing the challenge of providing access to the most remote facilities (the “last mile”). For the hardest-to-reach facilities in Zambia, we compared the cost of placing point of care (POC) viral load instruments at or near facilities to the cost of an expanded sample transportation network (STN) to deliver samples to centralized laboratories.

**Methods:**

We extended a previously described geospatial model for Zambia that first optimized a STN for centralized laboratories for 90% of estimated viral load volumes. Amongst the remaining 10% of volumes, facilities were identified as candidates for POC placement, and then instrument placement was optimized such that access and instrument utilization is maximized. We evaluated the full cost per test under three scenarios: 1) POC placement at all facilities identified for POC; 2)an optimized combination of both on-site POC placement and placement at facilities acting as POC hubs; and 3) integration into the centralized STN to allow use of centralized laboratories.

**Results:**

For the hardest-to-reach facilities, optimal POC placement covered a quarter of HIV-treating facilities. Scenario 2 resulted in a cost per test of $39.58, 6% less than the cost per test of scenario 1, $41.81. This is due to increased POC instrument utilization in scenario 2 where facilities can act as POC hubs. Scenario 3 was the most costly at $53.40 per test, due to high transport costs under the centralized model ($36 per test compared to $12 per test in scenario 2).

**Conclusions:**

POC VL testing may reduce the costs of expanding access to the hardest-to-reach populations, despite the cost of equipment and low patient volumes. An optimal combination of both on-site placement and the use of POC hubs can reduce the cost per test by 6–35% by reducing transport costs and increasing instrument utilization.

## Introduction

Viral load testing has been recommended by the World Health Organization as the optimal way to monitor patients on ART [1]. This has led to an acceleration of investment in viral load testing capacity in lower- and middle-income countries with viral load testing demand expected to increase from 14.7m tests in 2017 to 28.5m tests in 2022 [2]. Whilst viral load monitoring programs have been scaled up rapidly throughout Africa with investments to strengthen and coordinate sample transportation networks (STNs), it was estimated that only 44% of people on ART accessed routine viral load testing in 2016 in eastern and southern Africa [3], and rural areas generally report lower uptake of viral load monitoring services [4].

There is currently no formal, coordinated national transportation system for transporting blood samples from HIV treatment facilities to laboratories in Zambia, even for the largest urban healthcare facilities. Samples are largely transported on an ad-hoc basis organized by facilities using their own vehicles, motorbikes or public transport. Remote healthcare facilities face additional challenges: long distances to centralized laboratories, inadequate road infrastructure (exacerbated during the wet season), and the risk of blood sample degradation due to poor coordination, cold-chain failures, and unreliable transport. Since these remote and often low-volume facilities contribute just a minority of samples, little attention has been paid to how to integrate them into a STN. This is reflected in programmatic data from 3 provinces in Zambia which indicates that patients at low volume health facilities are less likely than patients at higher volume facilities to have a viral load sample tested due to challenges in drawing, storing, and transporting blood samples [5,6].

In a previously described analysis of the Zambian viral load monitoring programme, we developed a geospatial model that aimed to minimize the cost of a national viral load STN taking into account transport distances, driving times, and viral load blood sample demand at each facility [5]. In 2017, Zambia had nearly 800,000 patients on antiretroviral treatment (ART) at 1,475 facilities [7]. By 2020, with the rollout of the national ‘treat all’ guidelines, the number of patients on ART is expected to approach 1.2 million [8]. In the optimized STN previously described [5], access to viral load testing using 18 large, centralized labs could increase from 10% in 2016 to 91% in 2020, when it would reach a total of 800 HIV-treatment facilities with an estimated total viral load volume of approximately 1.5 million samples per year. Whilst this national STN ensures that the Ministry of Health’s 2020 target of 80% viral load volume coverage is efficiently achieved, it still leaves nearly one out of ten (9%) patients and nearly half (46%) of ART facilities without reliable access [9,10].

Mean transport cost per viral load sample transported in the modelled national STN was $2.10 per test. Although this is a modest share of the total cost of a viral load test (12%), sample transport costs are projected to increase substantially as Zambia scales up test access to the patient populations in the hardest to reach facilities, ultimately consuming up to 64% of the cost of a viral load test for the most remote 5% of patients [5,10]. These hardest-to-reach facilities could thus be candidates for point of care (POC) viral load instrument placement, eliminating sample transport costs and improving turn-around times for result delivery [11–14]. POC instruments aim to decentralize testing to the level of the facility. They are designed to be simple to use, with testing performed by lower cadres of healthcare staff, as well as to improve patient outcomes such as viral suppression and retention in care [15]. Though a number of studies have found POC technologies to be cost effective (both clinically and in terms of diagnostic performance) compared to conventional laboratory-based testing [16–21], most studies were conducted in urban, high volume facilities. How these POC technologies perform in low throughput remote settings and whether they remain cost-effective relative to conventional centralized laboratory testing is not well understood [22]. To explore this, we compared the *cost* of placing POC viral load test instruments at or near facilities in Zambia to the cost of an expanded STN to deliver samples to centralized laboratories for facilities not reached by the modelled national STN, as well as for the hardest-to-reach subset of these facilities.

## Methods

The overarching objective of our model was to identify and estimate the cost of strategies for optimizing viral load access for the most remote healthcare facilities (the “last mile”), which are a subset of those not reached through the modelled national STN. Access was defined as being able to conduct a viral load test on demand, either with a viral load POC testing device or by linking patient populations to a reliable and frequent sample transport service that ensures delivery of viral load samples to centralized laboratories.

### Study design

**Fig 1:**
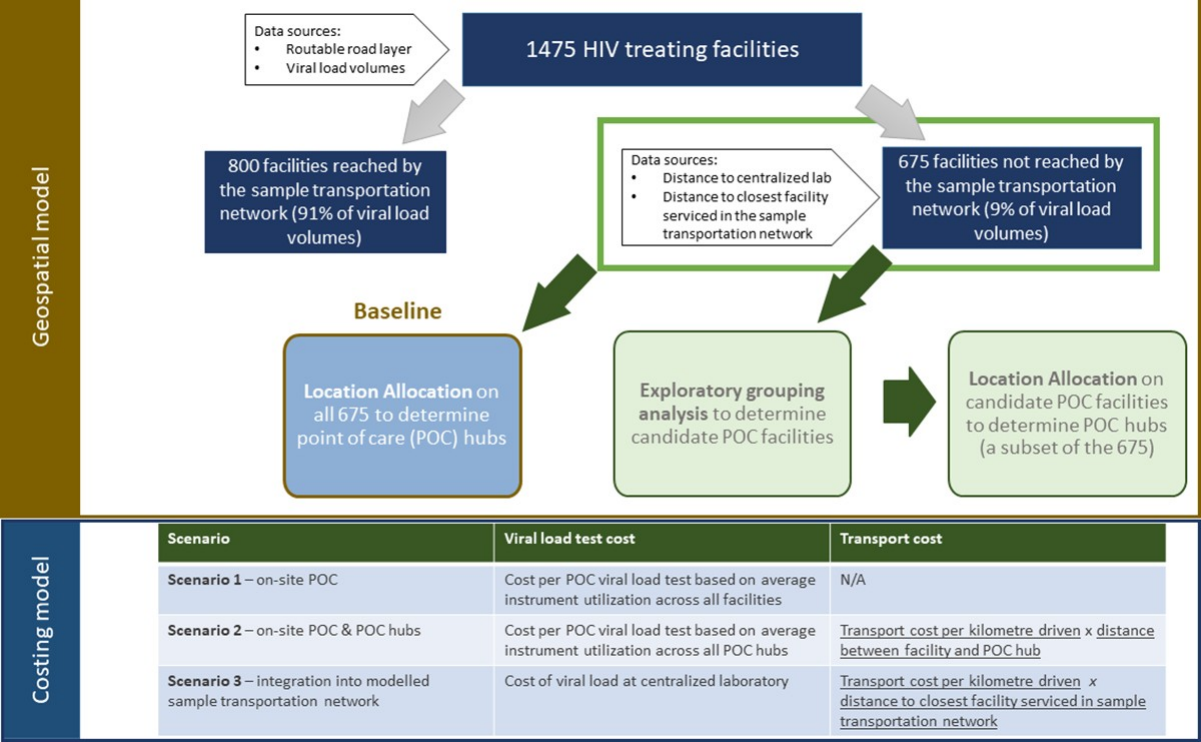
Schematic of the integration of the geospatial model with the cost model to determine the optimized placement of POC instruments.

We extended a previously described geospatial model that utilized a range of data to optimize the placement of POC viral load equipment at facilities not reached by the modelled national STN as well as a subset of the most remote facilities such that viral load patient coverage and instrument utilization (subject to transport constraints) are maximized [5]. *ArcGIS 10*.5 (*ESRI*, Redlands, California, USA) was used to run different algorithms to identify candidate POC facilities, select facilities for POC placement, and model the different scenarios described below (Table 1). The final geospatial model output was then included in a cost model to determine the total cost (test and transport) associated with each scenario (Fig 1**)**. Our analytical dataset included the 675 (out of 1475) HIV outpatient treatment facilities that were not reached through the modelled STN. For each site, the expected weekly viral load demand for 2020 for the patients at these facilities, the drive time to the nearest centralized laboratory, and the drive time to the nearest high volume facility were calculated. The expected weekly viral load demand for 2020 by facility was determined using patient volumes based on District Health Information System II (DHIS2) data on the number of patients on ART at the health facility level from March 2017 [7] and on provincial Spectrum modelling results[23] to project 2020 volumes as described previously [5]. The Global Positioning System (GPS) location of each facility as well as a routable road layer were used to calculate driving times and distances between facilities and laboratories [5].

**Table 1:** Modelled scenarios.

Scenario	Description	Facilities	
		Baseline	Candidate POC facilities
**Scenario 1**: True POC (on-site POC at all candidate facilities)	On-site POC placement at all facilities	Scenarios based on all 675 facilities not reached by the modelled national STN	Scenarios based on a subset of the 675 facilities not reached by the modelled national STN but identified as candidate POC facilities using exploratory cluster analysis.
**Scenario 2**: on-site POC and POC hubs	An optimized combination of both on-site POC placement and placement at facilities that would act as POC hubs for the facilities in their immediate surroundings. This scenario uses a Location Allocation model to identify POC hubs.		
**Scenario 3**: Expanded centralized sample transport network	Integration of the facilities into the centralized modelled national STN to allow use of centralized labs		

### Modelled scenarios

We estimated the full cost (test and transport) per test completed under three scenarios where we considered both POC placement at or near facilities and integration of facilities into an expanded STN for all 675 facilities that were not reached by the modelled national STN. We also estimated costs for a subset of these facilities that were deemed ideal candidates for POC placement due to their locations and volumes (Table 1**)**. An illustration of the model is provided in Fig 1.

### Exploratory cluster analysis

To identify the candidate facilities for POC placement, exploratory cluster analysis was conducted on the 675 facilities using ArcGIS’s Grouping Analysis tool. This tool uses a K-means algorithm to find a solution where facilities are grouped together such that attributes within one group are as similar as possible, and all groups are as different as possible[24]. The attributes used to distinguish similarities and differences were the drive times between the facility and the closest centralized laboratory and high volume facility and the weekly viral load volumes. Proximity to a centralized laboratory and/or to a high volume facility was used to distinguish facilities that would be considered ‘remote’ and hard to access from those with better access to testing facilities and organized transport routes. A two-hour drive time was chosen as a benchmark for access as it ensures time for the driver to collect samples, deliver the samples and return to the facility in the same working day. As stakeholders indicated, moreover, the main form of sample transport at these more remote facilities is motorbikes. Two hours drive time to a testing facility is thought to be the upper-bound of what is considered safe and appropriate for a motorbike driver. The optimal number of groups was determined using multiple iterations of the analysis and the Calinski-Harabasz pseudo F-statistic. Spatial constraints were not used to constrain group membership. Groups with low viral load volumes and large distances from a centralized laboratory or high volume facility were identified as candidates for POC placement.

### Location allocation for POC site selection

Both the baseline facilities (675 not reached by the modelled STN), and the facilities identified as candidates for placement of a POC instrument through the grouping analysis were then used as input into a location allocation model in order to determine POC hubs in scenario 2. This model aimed to optimally locate facilities for placement of a POC device whilst ensuring all facilities have access to a testing facility. Within the ArcGIS Network Analyst Extension, the Location Allocation solver uses a heuristic process to solve for a solution whereby facilities for POC placement are located such that all the demand is allocated to a POC device within a specified drive time (90 minutes in this instance) and coverage of facilities is maximized [25,26]. The algorithm for location allocation strives to place POC instruments that maximize ART facility coverage whilst minimizing the impedance factor, which is drive time, and as such incorporates facilities with lower cost of access first. Determining the number of facilities for POC placement is an iterative process, with more POC devices being allocated until all viral load demand is covered.

#### Existing and potential viral load testing instruments

Viral load testing is currently centralized at 18 laboratories across Zambia. Current equipment being used includes the Roche Cobas®Ampliprep/Cobas®TaqMan 48 and the Roche Cobas®Ampliprep/Cobas®TaqMan 96 (Roche Molecular Diagnostics, Branchburg, US). POC and near-POC equipment for viral load testing has not yet been approved for use in Zambia. For this analysis, we assumed procurement and use of the GeneXpert® Omni molecular diagnostic system, though not yet launched (Cepheid Inc. Sunnyvale CA, USA), as an example of a low output device. It has been designed for low output resource-limited settings, weighs only 1kg, has an integrated rechargeable battery providing 4 hours of power supply and a supplemental rechargeable battery supply with up to 12 hours of battery life, and has a weekly viral load capacity of approximately 30 tests. Tests are performed on plasma samples. Mini centrifuges were allocated to each POC instrument for centrifuging plasma samples prior to testing.

### Cost inputs

Costs included were the cost per kilometer driven (as per the Zambian official vehicle reimbursement rate), driver salaries, the capital cost of a motorbike, the all-inclusive cost per viral load test conducted at a centralized lab, and the cost of a viral load test using POC (Table 2). The cost per viral load test was estimated using the ‘Testing Platform Cost Model’ (TPCM) developed by HE^2^RO (http://www.heroza.org/researchtools/testing-platform-cost-model/). This costing model includes information on staff time and costs, materials required to conduct the test, platform costs, capital costs, overhead and shared costs, and related equipment (Table 2, S1 File).

**Table 2:** Key cost parameters and related assumptions.

Parameter	Estimate	Range	Sources and assumptions
**Transport costs**			
Vehicle running costs ($USD/km) *	$0.55	$0.51 - $0.59	Ministry of Health Reimbursement Rate
*Diesel price ($USD/litre)*	$1.24	$1.16-$1.33	http://www.globalpetrolprices.com/Zambia/diesel_prices/
Motorbike capital cost	$4,012	$4,000-$5,000	USAID procurement 2017, Zambia
Motorbike write-off period	4	3–5	Estimate
Allocation of capital to sample collection	1/5 (20%)		Motorbike will be required for one day a week to do sample collection
Monthly driver salaries	$432	$324-$540	Ministry of Health salary scales. Assumed a driver will be required one day a week to do sample collection
**Test costs**			
Centralised lab viral load test cost**	**$17**.**22**	**$15**.**00 - $20**.**06**	**Testing Platform Cost Model**
*Materials*	$14.74	$13.27 - $16.22	Biogroup Zambia Limited and Medical Stores Limited Catalogue 2016.
*Equipment costs*	$0.88	$0.43-$1.80	Procurement invoices, Centre for Infectious Disease Research in Zambia (CIDRZ) laboratory. Roche Cobas® Ampliprep/ Cobas® TaqMan 96 procured at $160,000.
*Staff*, *quality control*, *overhead*, *training and overhead costs*	$1.60	$1.30- $2.05	Government of Zambia salary scales. CIDRZ laboratory invoices, Lusaka; Medical Stores Limited Catalogue 2016; Discussions with the CIDRZ laboratory senior staff.
**POC viral load test cost** **(Utilization %)**	**$23**.**23** **(50%)**	**$20**.**78 - $263**.**73** **(100% - 1%)**	**Testing Platform Cost Model**
*Materials*	$17.27	$15.54-$19.00	Used the ceiling price for the viral load assay test kit (< 500,000 tests per year).[27]
*Equipment costs*	$2.18	$0.84-$132.20	Omni instrument cost estimate of $5000 based on report.[28]
*Staff*, *quality control*, *overhead*, *training and overhead costs*	$3.78	$2.19-$138.29	Government of Zambia salary scales; Kanyama clinic, Lusaka; Correspondence with district and facility staff. [27,29]
Max capacity of a POC platform per week (per day)	30 (6)		Test takes 90 minutes, assume 8 hour day, 5 days a week

*Zambian reimbursement formula is: ((Fuel Price*1.1)/2.5)

**We assume that the cost of a viral load test at the centralized lab does not vary with utilization as the addition of the hard to reach facilities’ volumes are too small to impact on test price (+/- $0.01).

### Cost per viral load test conducted

For scenario 1, the all POC scenario, the cost per test was estimated based on the average instrument utilization across the facilities. Sample transport costs were assumed to be zero for facilities allocated a POC device. Similarly, for scenario 2, which includes both on-site POC and POC hubs, the cost per test was estimated based on average instrument utilization. For facilities utilizing POC hubs, the distance between the facility and the POC hub was used to estimate the transport cost. For scenario 3, the transport cost of integrating these facilities into the modelled national STN was calculated using an origin-destination cost matrix which determines the distance between the identified facility and the nearest facility that is serviced by the modelled STN.

A sensitivity analysis on cost inputs (doubling and tripling the cost of POC equipment; reducing the price of a viral load test conducted in a centralized laboratory by 20%; reducing the fixed transport trip cost component by 50%), as well as adjusting the failure rate on a POC equipment to take into account test failures, electricity outtages and the use of lower level staff cadres, was conducted to determine the impact of these parameters on our results.

## Results

Using exploratory cluster analysis, 337 facilities of the 675 facilities not reached by the modelled STN were identified as ideal facilities for POC instrument placement, representing nearly a quarter (23%) of all Zambian HIV treatment facilities. These 337 facilities will jointly require 1,056 viral loads per week in 2020, representing 3.2% of total national viral load volumes (Table 3). The remaining 50% of the unreached facilities (338/675) were either not considered ‘remote’ (i.e. they have better access to testing facilities and organized transport routes because they are within a two-hour drive to the nearest testing facility) or they had volumes in excess of the capacity of a GeneXpert Omni and were assumed to be serviced more efficiently by higher capacity equipment (for example, the Cepheid GeneXpert® IV) than is reflected in this analysis. This is corroborated by the lower transport costs to integrate the 338 facilities that were not identified as candidates for POC into the centralized system ($10.30 per viral load) compared to the sample transport costs of the 337 facilities identified as candidates for POC ($36.22 per viral load).

**Table 3:** ART facilities and POC candidate facilities.

	Number of facilities (% of total)	Weekly viral load volume (% of total)
Total ART facilities	1475 (100%)	32,800 (100%)
Facilities reached by modelled STN	800 (54%)	29,842 (91%)
Facilities not reached (Baseline)	675 (46%)	2,957 (9%)
Identified potential POC candidates	337 (23%)	1,056 (3.2%)

**Fig 2:**
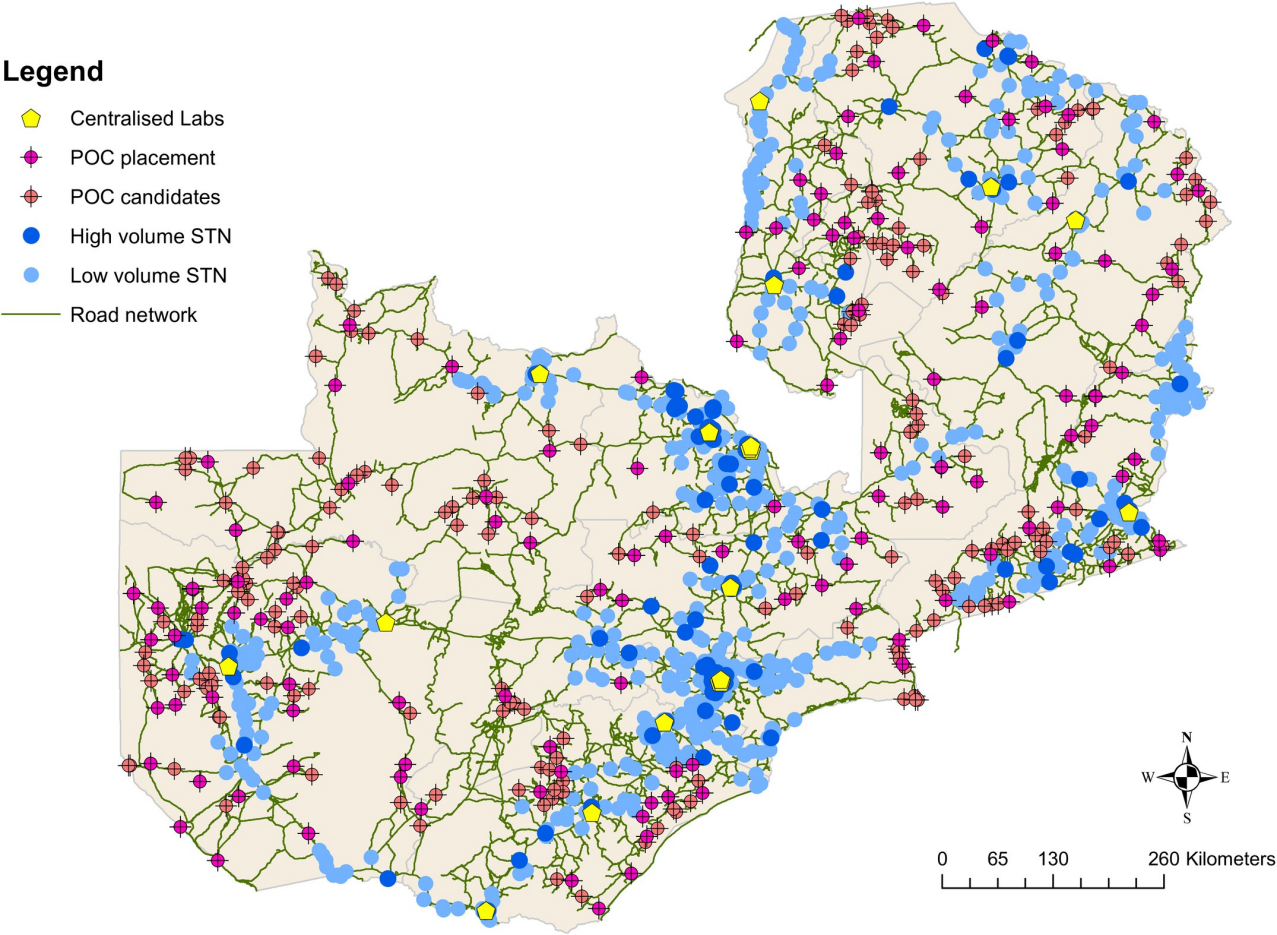
Illustration of the location of the 337 POC candidates (pink) relative to centralized laboratories, facilities reached through the STN and the unreached facilities that were not considered candidates for POC instrument placement.

Both the identified 337 POC candidate facilities and the 675 baseline facilities were then used as input into a location allocation model that optimally located facilities for placement of a POC device by maximizing POC testing coverage to ensure all facilities have access to a testing facility once a week. For the on-site and POC hub scenario (scenario 2), 133/337 of these facilities were allocated POC devices and functioned as POC hubs to the facilities in their near surroundings (Fig 2), and 208/675 facilities were allocated POC devices in the baseline scenario.

The baseline case that included all 675 facilities that were not included in the modelled national STN, showed modest cost differences between scenarios. Scenario 1, which involved allocating a POC instrument to each of the 675 facilities, resulted in a cost per test of $35.79. The cost per test under scenario 2 was slightly lower at $34.76, and the cost per test under the centralized scenario 3 was the highest at $36.78. These results were accentuated when the analysis was restricted to the subset of the hardest-to-reach facilities (377). Although all scenarios were modelled to reach the same 337 facilities, the resources required to reach these facilities differed sharply between them (Table 4). Scenario 1, which involved allocating a POC instrument to each of the 337 facilities, resulted in a cost per test of $41.80. The cost per test in scenario 2 was $39.58, and the cost per test in scenario 3 was the highest at $53.40. Scenario 2 outperforms scenario 1 due to the increased POC instrument utilization when an instrument can act as a POC hub for nearby facilities, with average instrument utilization of 26%, compared to only on-site POC which has an average instrument utilization of just 10%. Scenario 3 is the most costly due to high transport costs under the centralized model ($36 per test compared to $12 transport cost per test in scenario 2 for the most remote 337 facilities). The cost per test under scenario 2 is 6% and 35% lower than under scenario 1 and scenario 3, respectively.

**Table 4:** Cost per viral load test by scenario for the hardest-to-reach patients.

	Scenario 1 (on-site POC at all facilities)	Scenario 2 (on-site POC and POC hubs)	Scenario 3 (expanded sample transport network)
**Baseline (675 facilities)**			
Median total cost per test (IQR)	$35.79 ($33.91 - $37.86)	$34.76 ($33.38 - $36.69)	$36.78 ($35.49 - $38.78)
**Candidate POC facilities (377)**			
Median total cost per test (IQR)	$41.81 ($39.62 - $44.17)	$39.58 ($37.95 - $41.76)	$53.44 ($52.08 - $55.68)
*Test (IQR)*	$41.81 ($39.62 - $44.17)	$27.94 ($26.47 - $29.64)	$17.22 ($16.11 - $18.64)
*Sample transport (IQR)*	$0	$11.64 ($11.56 - $11.88)	$36.22 ($36.10 - $36.63)
Total cost of scenario (IQR)	$2 296 680 ($2 176 106 - $2 426 044)	$2 174 007 ($2 084 701 - $2 294 193)	$2 935 558 ($2 860 924 - $3 058 393)
Savings from using optimized POC and near-POC	$122 673	-	$761 551

For the hardest-to-reach facilities, scenario 2 costs on average $2.2m per year to implement whilst the expanded centralized STN costs on average $2.9m (scenario 3). Scenario 2 could thus save the government of Zambia $762,000 per year, or 26% of the cost of reaching these viral load volumes. This saving is primarily due to a reduction in transport costs. To put this into perspective, the total estimated transport cost of the entire previously modelled STN was $3.2m per year to reach 91% of the viral load volumes [5]. In comparison, the annual transport cost to reach just 3.2% of viral load volumes is $1.9m under scenario 3 (not shown), excluding the considerable costs of coordination and logistics management required to ensure 100% coverage. Thus, the cost of integrating the hardest to reach viral load patients into the centralized STN would result in a 60% increase on the cost of the entire STN.

The cost per viral load test was most sensitive to increasing the capital cost of POC equipment. However, only when capital costs were tripled did the true POC scenario 1 become more expensive ($56.86) than the centralized scenario ($53.44). Scenario 2 remained the least costly scenario even when the equipment cost was tripled from $5000 to $15,000 at $45.74 per viral load test. A reduction in the price of a viral load test conducted in a centralized laboratory by 20% reduced the cost of the scenario 3 by 6% to $50.00. Reducing the fixed transport trip cost reduced the cost of scenario 2 and 3 by 6–8% but did not change the conclusions. Finally, the failure rate of viral load testing on POC instruments needs to be 50% before scenario 2 became more expensive than scenario 3.

## Discussion

Many studies have found POC technologies to have high diagnostic accuracy [30,31] and be cost effective compared to conventional laboratory-based testing [16–20]. How these technologies perform in low throughput settings, what the strategies for scale-up of POC technologies are and whether they remain cost-effective relative to conventional centralized laboratory testing in terms of diagnostic accuracy or clinical impact, however, is not well understood [22,30]. By optimizing the allocation of POC technologies to reach the ‘last mile’ of viral load volumes, we have shown that costs can be reduced by as much as 35% for these viral loads compared to integrating these facilities into the centralized laboratory system, despite the cost of POC equipment and low patient volumes. We have shown that POC viral load technologies can play an important role in complementing, not replacing, conventional centralized testing platforms when placement decisions are informed by patient volumes, characteristics (such as remoteness), cost per test and instrument utilization [32].

Low utilization of POC instruments in the near-POC scenario accounts for 26% of the test cost per test. As such, further improvement of POC instrument utilization can significantly reduce the cost per test by this amount. Cepheid’s GeneXpert Omni, like many new POC technologies, is a polyvalent platform, allowing for the testing of a number of other tests. Sharing excess capacity with other tests will improve utilization and reduce the cost per test. Transport costs account for 68% of the cost per test under scenario 3, the centralized viral load testing scenario. The cost per test transported under the centralized model could also be reduced by the incorporation of other types of samples, such as full blood counts, TB tests, and other primary care investigations. The cost per test reported here is for a dedicated viral load blood STN. If samples for other purposes could share the transport cost, transport costs in both scenario 2 and 3 would be reduced, and a larger range of laboratory tests would become accessible.

To our knowledge, this is the first programmatic geospatial model that has developed a methodology for identifying candidate facilities for POC viral load placement, optimizing POC instrument placement and then comparing the test and transport costs of a POC model to a centralized model. The study is also unique in terms of its use of a rich dataset: a routable road network containing wet season drive time data between all facilities and laboratories, as well as laboratory and facility location data matched to programmatic ART data. This allows for a highly accurate calculation of the costs for all scenarios. Whilst this study has relied on a highly detailed road network layer, it is possible to replicate this geospatial framework in other countries using open source road networks (e.g. Open Street Maps) as well as proprietary data sources.

There are several limitations to our approach. Using our previous analysis as a starting point, we have not modelled a complete tiered laboratory network solution for Zambia [5,11,12]. This analysis is focused on those facilities not served by the modelled STN, and more narrowly, on the hardest-to-reach, most costly, last-mile facilities. Second, we restricted our analysis to a single, very low-output viral load POC device. Different POC viral load technologies will have different costs and different infrastructure requirements, as well as different capacities and as a result utilization. However, even if a different technology were chosen, it would need to be more than three times more expensive before it would be out performed by centralized testing. Third, considerations for electricity requirements, staff capacity, reagent transport, cold storage and connectivity were not taken into account when determining POC candidates [33]. Whilst continuous power is not important given the battery abilities of the POC Omni, it might be required for centrifuging and plasma storage. However, even if these factors contributed to a failure rate of 50%, the POC scenarios would still outperform the centralized scenario. Fourth, our results are conservative, as the clinical benefits of POC in terms of improved turn-around time or result delivery on the HIV treatment cascade has not been quantified. If the outcome metric was the cost per test delivered to the patient, the cost per test under scenario 3 may increase to $64, representing a 63% increase on the cost per outcome in scenario 2– making the POC scenarios even more attractive. This is assuming that there is a lower probability under the centralized STN that results will actually make it back to the patient (for example, 83% delivery rate for centralized testing [34] compared to nearly 100% in the POC scenario). Lastly, this analysis explored the placement of POC instruments at the hardest-to-reach facilities as a means to facilitate the expansion of viral load access. It has not attempted to compare this strategy to other strategies that facilitate the expansion of viral load access, for example, the use of dried specimens or drones [6,35,36].

## Conclusions

In conclusion, by optimizing the transport network and optimally placing POC instruments, the additional costs that countries and funders will incur for scaling up viral load testing to the last mile will be partially offset by more efficiency in transport and platform utilization. These results may be generalizable across a broad number of sample and test types and throughout other African countries that have large areas of land with low population density and are seeking strategies to increase access to rural populations [31,37]. If experience demonstrates the feasibility and sustainability of this combined approach—adding POC and near-POC instruments to reach the most remote facilities—then it may be an important complementary strategy to conventional centralized testing in a national viral load program.

## Supporting information

S1 File Supplementary material. Microsoft word document. Includes sources and calculations related to the costing of centralized and decentralized viral load testing.
(PDF)

S2 File Data. Excel document. Includes data underlying the findings included in this manuscript.
(XLSX)
