# Supplementary material for: Optimizing viral load testing access for the last mile: Geospatial cost model for point of care instrument placement
Source: PLoS One. 2019 Aug 26;14(8):e0221586. doi: 10.1371/journal.pone.0221586 (PMC6709899; doi:10.1371/journal.pone.0221586)
Supplement: S1 File — Microsoft word document. Includes sources and calculations related to the costing of centralized and decentralized viral load testing. (PDF) [file pone.0221586.s001.pdf]

Supporting Information

S1 File. Viral load costing

The cost per viral load test for both a centralised test setting and a point of care instrument was estimated using the Testing Platform Cost Model (TPCM) developed by HE^{2}RO staff (www.heroza.org). This cost model provides a simple tool for analysts and decision-makers to determine the cost per test for a testing platform from a provider's perspective. The cost to a provider of a test depends on many individual components. These include the material costs per test, the salaries of staff who conduct start-up and daily quality control activities on the machine, the salaries of staff who perform the test, how the technology is integrated into patient flow and management, equipment and other related costs (e.g. insurance, replacement parts), the expected working life of the platform itself, and the opportunity cost of funds used to acquire the platform (i.e. the discount rate).

This tool is used to calculate the cost per test in two scenarios: (1) a viral load test conducted at a large central referral laboratory using a bottom-up costing approach, and (2) a viral load test on a point of care device in a small rural healthcare facility using an ingredient's based costing approach. Shared costs, in the case of the large centralised referral laboratory, are allocated according to the proportion of laboratory tests that the test under evaluation accounts for. Overhead costs are allocated according to the space allocated to the specific test under evaluation relative to the entire facility.

For both the centralised viral load test cost and the point of care test cost, the inflation rate was based on data from the Zambian Central statistical Office (Zambia Data Portal) and the exchange rate between the Zambian kwacha and the United States dollar was based on data from the Bank of Zambia. All costs are reported in US dollars and for 2018. Sample collection costs were not included in this cost per test as they should not differ between the two scenarios (centralised and point of care test costs) as they both rely on whole blood sample collection.

Centralised test cost at a large centralised lab in Lusaka, Zambia

For this paper, we based our cost estimates for a viral load conducted at a centralised laboratory on a bottom-up costing analysis conducted at the Centre for Infectious Disease Research in Zambia (CIDRZ) laboratory located in the Kalingalinga area of Lusaka, Zambia. It is the primary laboratory for most of Lusaka province and it serves as a reference laboratory for molecular diagnostics (HIV, DNA, PCR and viral load testing) for a number of neighboring provinces. It processes and conducts testing on approximately 1600 blood samples per day (of which 400 are viral loads). Viral load testing is conducted using the two Roche Cobas®Ampliprep/ Cobas®TaqMan 96 (CAP/CTM 96) (Roche Molecular Diagnostics, Branchburg, US). There are a number of shared activities across these blood sample tests as all blood samples are sorted, registered and stored together – as such all shared costs from these activities were allocated across all blood samples. All assumptions and sources are detailed in S1 Table below.

S1 Table. Centralised viral load cost per test – assumptions and sources

Cost category	Unit cost (US$)	Items included	Assumptions	Sources
Materials	14.74	Pipette tips; reagents (including SPU for cobas, Tip-k for Cobas ampliprep, S-tube input for Cobas, KIT CAP-G/CTM wash reagent)	Included the central Medical Stores Limited distribution margin of 1.5%.	Biogroup Zambia Limited and Medical Stores Limited Catalogue 2016
Staff	0.47	Laboratory staff activities for viral load testing included: registering the sample/data entry; sample preparation and running; interpretation of the result/result delivery. Other shared activities conducted by laboratory assistants on all blood samples included sample sorting, centrifuging, aliquoting, archiving, searching, temperature monitoring etc.	Activity time estimations based on discussions with staff at the CIDRZ laboratory as well as laboratory records. Shared staff time allocated across all blood sample tests.	Government of Zambia salary scales. CIDRZ laboratory, Lusaka
Quality control	0.04	Quality control activities conducted by a laboratory scientist every shift included start-up/equipment maintenance (including checking the buffer and control tubes, emptying waste). A cleaner also cleaned the room containing the viral load equipment every day.	Activity time estimations based on discussions with staff at the CIDRZ laboratory	Used Zambian government salaries for staff time. Medical Stores Limited Catalogue 2016; CIDRZ laboratory, Lusaka
Equipment	0.88	CAP/CTM 96 ($160,000), air-conditioners, refrigerators, fire extinguishers, computers, biosafety cabinets, chairs, freezers, tables generator, thermomixer, printers, uninterrupted power supply etc.	Estimated the working life of a CAP/CTM 96 (5 years); estimated other equipment working life based on the South African Revenue Service write-off periods as well as discussions with laboratory personnel. All equipment annualized at 5% discount rate. Shared equipment for the laboratory allocated across all blood sample tests.	USAID procurement invoices, other invoices, CIDRZ laboratory
Other	0.74	Includes annual and upfront training for laboratory personnel; maintenance and insurance costs for equipment (e.g. CAP/CTM 96 and fridges/freezers); waste removal; dinner and transport allowance for extra shift work.	Viral loads contributes 40% of all lab waste (based on CIDRZ staff estimate)	Used Zambian government salaries for staff time for training, used invoices from CIDRZ lab for maintenance, insurance, staff per diems, and waste removal
Overhead	0.35	Electricity, security services, motor vehicles, overhead staff (e.g. other laboratory scientists, managers and maintenance staff)	Estimated electricity based on EQUIP office electricity bill in Lusaka. Allocated 20.4% of the total building size to viral load testing activities (based on space used to conduct the test as well as a proportion of shared space).	Discussions with the CIDRZ laboratory senior staff.
**Total**	**17.22**			

Point of care test cost at a rural health centre or health post in Zambia

For this paper, we based our point of care viral load test costs on the GeneXpert® Omni molecular diagnostic system (Cepheid Inc. Sunnyvale CA, USA). This system is not yet available or in use in Zambia, and is only expected to be launched globally in 2019. As such, many costs and inputs were estimated. These are all detailed in S2 Table below. The hypothesized setting is a small, rural health centre or rural health post. The below estimates show the expected case with a utilization of 50% (or 3 tests per day). Point of care tests costs for different levels of utilization were calculated for the model by varying the number of tests conducted per day. This changed the per test cost for equipment, other, overhead and quality control cost categories.

S2 Table. Point of care viral cost per test – assumptions and sources

Cost category	Unit cost (US$)	Items included	Assumptions	Sources
Materials	17.27	Cepheid's Xpert HIV-1 viral load assay test kit (contains cartridges, reagents, disposable transfer pipettes, CD with ADF, PI.)	Used the ceiling price for the viral load assay test kit (< 500,000 tests per year).	[1]
Staff	1.05	Nursing staff activities included: centrifuging the whole blood sample, preparing the specimen, preparing the cartridge by transferring 1 ml of plasma into the sample chamber using the transfer pipette, and loading the cartridge into the Omni.	Activity time estimations based on discussions with study personnel at Kanyama clinic in Lusaka (using the Alere q for EID).	Government of Zambia salary scales. Kanyama clinic, Lusaka
Quality control	0.56	Quality control activities by a nursing sister included cleaning and switching on the Omni, switching it off at the end of the day, uploading results to the server, and a daily wipe-down of the instrument.	Activity time estimations based on discussions with study personnel at Kanyama clinic in Lusaka (using the Alere q for EID).	Government of Zambia salary scales. Kanyama clinic, Lusaka
Equipment	2.18	Cepheid Omni, printer, inverter and mini centrifuge	Estimated the cost of a Cepheid Omni, and working life ($5000, 5 years). All equipment annualized at 5% discount rate.	Omni instrument cost estimate based on [2]; other equipment prices based on invoices.
Other	1.86	Includes annual training of 3 hours; paper for printing results; warranty and service maintenance plan.	Costs for the warranty and service maintenance plan were assumed to be 50% of a GeneXpert II.	Training time and warranty [1]; service maintenance plan [3]; paper for printing (invoice)
Overhead	0.3	Facility government grant; electricity; overhead staff	Assumed 1 cleaner, 1 handyman and 1 nurse in charge as overhead staff at a government small rural clinic. Allocated 1% of the total building size to the Omni activities.	Correspondence with district and facility staff.
**Total**	**23.23**			

References

1. Medecines Sans Frontiers. Putting HIV and HCV to the test. A product guide for point-of-care CD4 tests and laboratory-based and point-of-care HIV and HCV viral load tests. 3rd edition. In: MSF Access [Internet]. 2017 p. 112. Available: https://issuu.com/msf_access/docs/hiv_report_puttinghivhcvtothetest_e
2. Medecines Sans Frontiers and Ministry of Health of the Krygyz Republic. Treating Patients, Not Disease: People-Centered Approach. 2018. Available: http://msf-tb-symposium.org/files/1715/2023/2923/1.1_Kathleen_England_TB_Diagnostics_ENG.pdf
3. The Global Fund. HIV Viral Load and Early Infant Diagnosis Selection and Procurement Information Tool [Internet]. 2017 [cited 1 Mar 2018]. Available: https://www.theglobalfund.org/media/5765/psm_viralloadearlyinfantdiagnosis_content_en.pdf
